# An Evaluation of Hemostatic Abnormalities in Patients With Hemophilia According to the Activated Partial Thromboplastin Time Waveform

**DOI:** 10.1177/1076029618757344

**Published:** 2018-02-13

**Authors:** Haruna Katayama, Takeshi Matsumoto, Hideo Wada, Naoki Fujimoto, Junki Toyoda, Yasunori Abe, Kohshi Ohishi, Yoshiki Yamashita, Makoto Ikejiri, Koji Habe, Naoyuki Katayama

**Affiliations:** 1 Department of Hematology and Oncology, Mie University Graduate School of Medicine, Tsu, Mie, Japan; 2 Department of Ketsueki-Gyouko, Ogikubo hospital, Suginami, Tokyo, Japan; 3 Division of Blood Transfusion Medicine and Cell Therapy, Mie University Graduate School of Medicine, Tsu, Mie, Japan; 4 Department of Molecular and Laboratory Medicine, Mie University Graduate School of Medicine, Tsu, Mie, Japan; 5 Central Laboratory, Mie University Graduate School of Medicine, Tsu, Mie, Japan; 6 Department of Dermatology, Mie University Graduate School of medicine, Tsu, Mie, Japan

**Keywords:** APTT, waveform, biphasic waveform, hemophilia, lupus anticoagulant

## Abstract

The usefulness of the waveform of activated partial thromboplastin time (APTT) in various diseases has been evaluated in recent years. The APTT waveform was examined in patients with hemophilia and patients positive for lupus anticoagulant (LA). The correlation with the FVIII activity was highest for the height of acceleration peak. The peak time of acceleration, velocity, and ½ fibrin formation, and the width of acceleration and velocity were significantly long and the height of acceleration was significantly low in patients with hemophilia. The height of velocity was significantly low in patients with hemophilia with inhibitor. There were no significant differences in the APTT waveform between patients with hemophilia and patients with LA, but the peak of acceleration and ½ fibrin formation were significantly longer and the height of acceleration and velocity were significantly lower in patients with hemophilia with inhibitor than in the patients with LA. Wave changes in the APTT were observed in all 22 patients positive for LA, while a biphasic waveform was observed in patients with hemophilia with FVIII activity <10.0%. The APTT waveform is useful for the analysis of hemostatic abnormalities in patients with hemophilia.

## Introduction

The activated partial thromboplastin time (APTT) and prothrombin time (PT) are generally used as routine laboratory clotting time assays, however, they only show the time. Activated partial thromboplastin time is useful for monitoring heparin treatment^1^ and for diagnosing the presence of lupus anticoagulant (LA)^2^ and coagulation factor deficiency in the intrinsic pathway such as hemophilia and acquired hemophilia A (AHA).^3^ Following the implementation of manual measurement, an automatic optical coagulation analyzer was recently developed, facilitating the performance of multiple assays. In addition, optical end-point coagulation analyzers can visualize the clot reaction curve as the APTT.

In previous reports, an abnormal biphasic curve of the APTT waveform was reported to be associated with the early detection of disseminated intravascular coagulation^4,5^ using Platelin LS APTT reagent with the MDA II (Organon Teknika, Cambridge, United Kingdom) analyzer. The ACL-TOP analyzer for APTT using APTT-synthetic phospholipids (SPs) is able to display the clot reaction curves and automatically calculates the absorbance data to display the first and second derivative curves (DCs) using the manufacture’s software program (Instrumentation Laboratory, Bedford, Massachusetts). The first and second DCs reflect the velocity and acceleration, respectively, at various points throughout the clotting reaction.^6^ Evaluations of the first and second DCs in the APTT waveform are reported to be useful for detecting coagulation factor deficiency and the presence of coagulation inhibitor,^7,8^ as well as for monitoring anticoagulant treatment.^9^ The differential diagnosis among hemophilia, AHA, and patients positive for LA^3,8,10^ is difficult to make based solely on the results of a clotting time assay. In addition, it is difficult to monitor the direct oral anticoagulant level, which is monitored by anti-Xa assays, using a clotting assay.^9,11,12^

In this study, we measured and analyzed the APTT waveform in patients with hemophilia with and without inhibitor, those with AHA, those with von Willebrand disease (VWD), those positive for LA, and those treated with warfarin to examine the relationship between the hemostatic abnormalities and the parameters of the APTT waveform.

## Materials and Methods

An APTT assay was performed with plasma samples from 30 healthy volunteers (10 females and 20 males; median age, 21 years; 25-75 percentile, 20-24 years), 22 patients with hemophilia negative for inhibitor (median age, 58 years; 42-64 years), 6 patients who were positive for FVIII inhibitors (median age, 61 years; 44-67 years), 3 patients with AHA (67 years; 61-74 years), 6 patients with VWD (58 years; 35-69 years), 22 patients who were positive for LA (18 females and 4 males; median age, 60 years; range, 40-73 years), and 20 patients who were treated with warfarin (15 females and 5 males; median age, 62 years; 56-70 years). The samples were obtained at Mie University Hospital. The patients with hemophilia included 17 patients with hemophilia A and 5 with hemophilia B; most of the patients with hemophilia were prophylactically treated with FVIII or FIX concentrate.

Routine APTT and FVIII activity was measured using CS 2500 (Sysmex, Kobe, Japan) with thrombocheck APTT (Sysmex) and thrombocheck FVIII (Sysmex). The LA status was determined based on the diluted Russell viper venom time.^10^ Thirteen of the 22 patients with LA had thrombotic complications. The PT of the patients who were treated with warfarin was controlled to keep it within the range of 1.6 to 2.6, according to the international normalized ratio.

The study protocol was approved by the Human Ethics Review Committee of the Mie University School of Medicine and written informed consent was obtained from each of the participant. This study was faithfully carried out in accordance with the principles of the Declaration of Helsinki.

**Figure 1.**
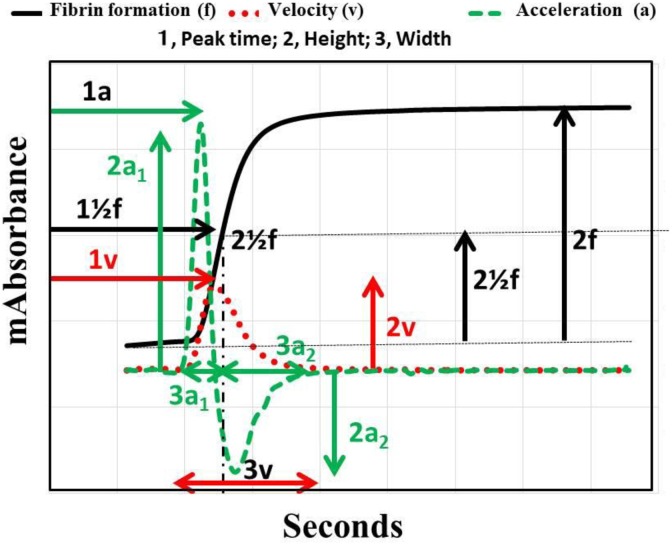
Activated partial thromboplastin time (APTT) waveform. a indicates acceleration; v, velocity; ½f, ½ fibrin formation; 1, peak time; 2, height; 3, width; 1a, peak time of acceleration; 1v, peak time of velocity; 1_1/2_f, peak time of ½ height of fibrin formation; 2a_1_, height of acceleration peak 1; 2a_2_, height of acceleration peak 2; 2v, height of velocity; 2_1/2_f, height of ½ fibrin formation; 3a_1_, width of acceleration peak 1; 3a_2_, width of acceleration peak 2; 3v, width of velocity, bp, biphasic waveform.

The APTT was measured using the APTT-SP, which use silica as an activator of FXII and SPs (Instrumentation Laboratory), on ACL-TOP system. Three types of curves are shown in the monitor of the ACL-TOP system (Figure 1). One is a curve showing the changes in the absorbance observed while measuring the APTT, corresponding to the fibrin formation. The second is the first derivative of the absorbance, corresponding to the coagulation velocity. The third is the second derivative of the absorbance, corresponding to the coagulation acceleration. For the waveform analysis, we first checked the presence of an abnormal curve showing a biphasic waveform on the first and/or second DCs. Furthermore, as shown in Figure 1, we calculated the following 10 parameters on the first or second DC by manual handling of the mouse: 1a_1_, acceleration; 1v, velocity; 1½f, time for ½ the height of fibrin formation; 2a_1_, the height of the acceleration peak 1; 2a_2_, the height of the acceleration peak 2; 2v, the height of the velocity; 2½f, the height of the fibrin formation; 3a_1_, the width of the acceleration peak 1; 3a_2_, the width of the acceleration peak 2, and 3v, the width of the velocity.

The APTT waveform assay was also performed in 1% or 10% of coagulation factor–deficient plasma using normal pooled plasma and HemosIL FII-, FV-, FVII-, FVIII-, FIX-, FX-, FXI-, or FXII-deficient plasma (Instrumentation Laboratory).

### Statistical Analyses

The data are expressed as the median (25th-75th percentiles). The differences between the groups were examined using the Mann-Whitney *U* test. *P* values of <.05 were considered to indicate statistical significance. All of the statistical analyses were performed using the Stat Flex software package (version 6; Artec Co Ltd, Osaka, Japan).

## Results

**Figure 2.**
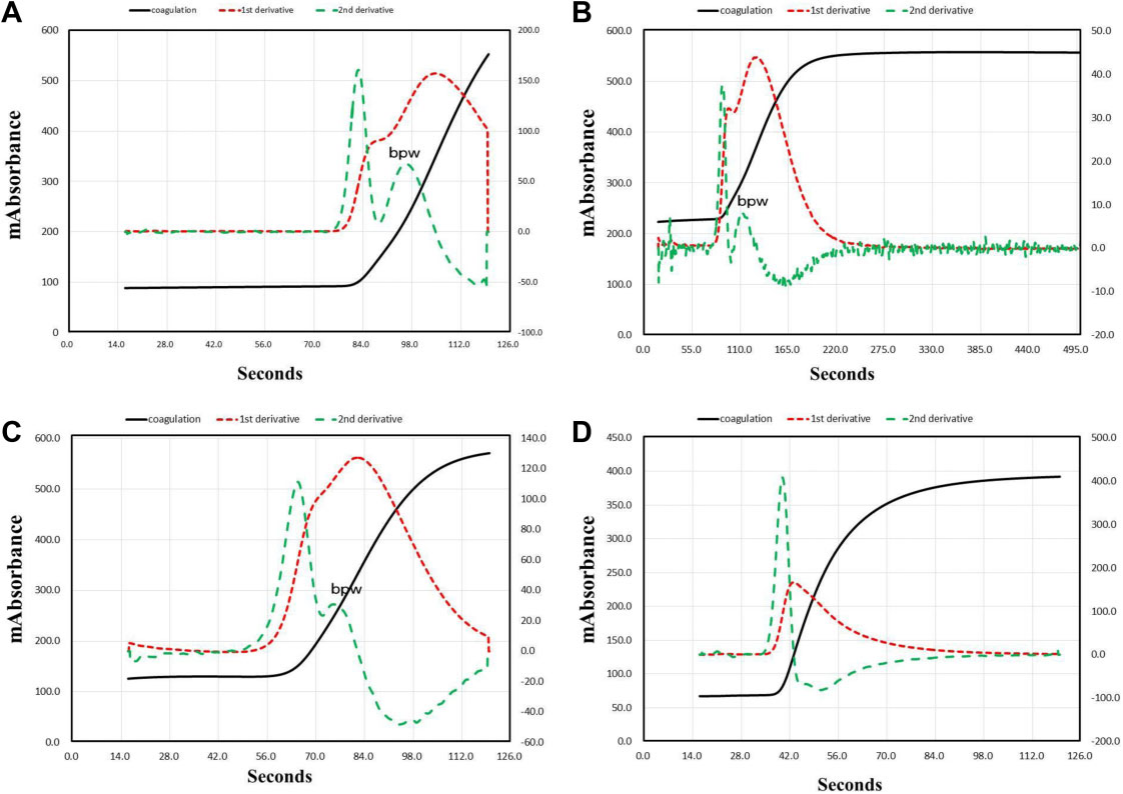
Activated partial thromboplastin time (APTT) waveform in a patient with hemophilia without inhibitor (A), with inhibitor (B), a patient positive for LA (C), and a patient treated with warfarin (D).

**Figure 3.**
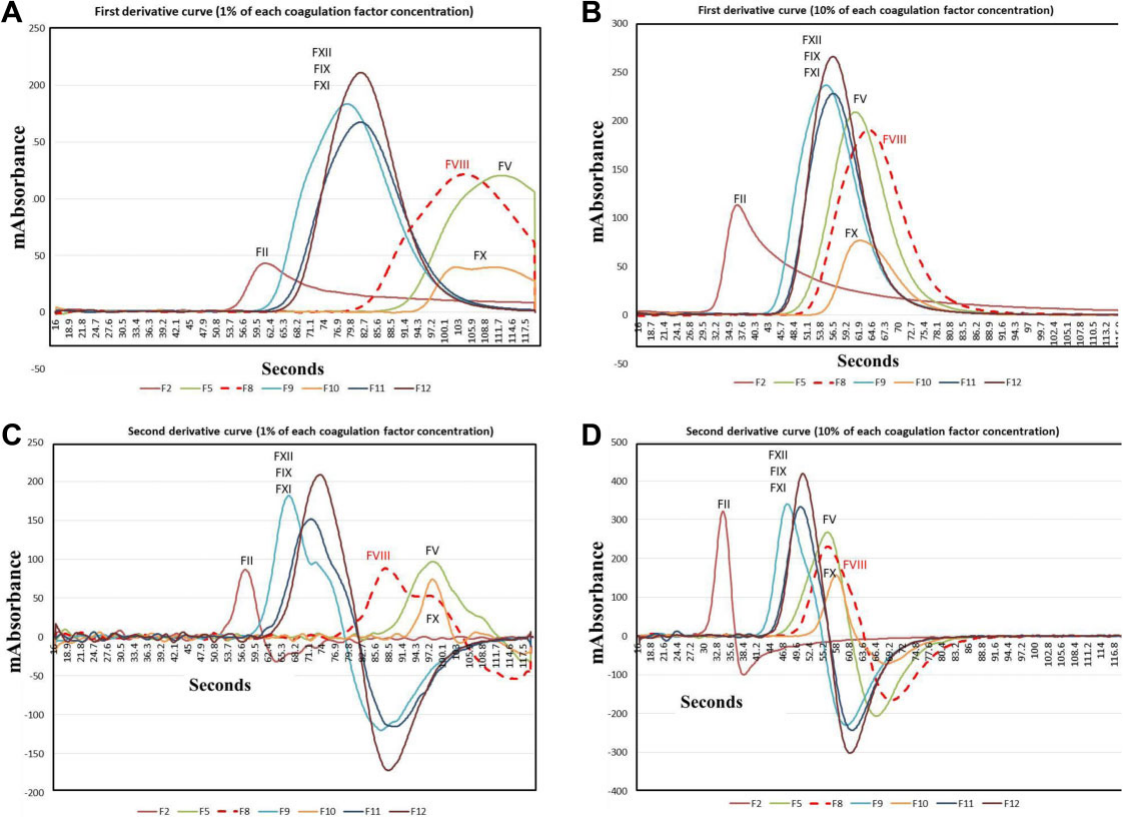
Velocity (A, B) and acceleration (C, D) curves in plasma deficient for various coagulation factors at 1% (A, C) and 10% (B, D).

Figure 2 shows the APTT waveform of hemophilia A without and with inhibitor along with patients positive for LA and patients treated with warfarin. A biphasic waveform was observed in patients with hemophilia A with and without inhibitor and patient positive for LA. The peak time of acceleration, velocity, and ½ fibrin formation was significantly longer in patients with hemophilia with and without inhibitor and in patients positive for LA than in healthy volunteers. The height of acceleration and velocity was significantly lower in patients with hemophilia with inhibitor than in healthy volunteers. Figure 3 shows the velocity and acceleration curves in 1% and 10% plasma deficient in each coagulation factor. The peak time was significantly longer in the FV-, FVIII-, and FX-deficient plasma than normal plasma, and the height of velocity was the lowest in FII- and FX-deficient plasma. The biphasic wave was observed in 1% FVIII-deficient plasma, but not in 10%.

The correlation with the FVIII activity (Table 1) was highest for the height of acceleration peak-1 and peak-2 (*r* = .760, *P* < .0001, respectively), and the correlation was similar among the peak of acceleration, peak of velocity, height of velocity, peak of ½ fibrin formation, and APTT. However, the correlation with the FVIII activity was the lowest for the fibrinogen level. The peak of acceleration, velocity, and ½ fibrin formation, and width of acceleration-1 and acceleration-2 and velocity were significantly longer in patients with hemophilia with or without inhibitor than in healthy volunteers (Table 2). The height of acceleration-1 and acceleration-2 was significantly lower in patients with hemophilia with or without inhibitor than in healthy volunteers, but the height of ½ fibrin formation was significantly higher in patients with hemophilia patients with or without inhibitor than in healthy volunteers. The height of velocity was significantly lower in patients with hemophilia with inhibitor than in healthy volunteers, but there were no significant differences between healthy volunteers and patients with hemophilia without inhibitor. There was no significant difference in most parameters of the APTT waveform aside from the width of acceleration and velocity between healthy volunteers and patients with VWD.

**Table 1.** Relationship Between FVIII Concentration and Parameters of APTT Waveform.

	Correlation Coefficient	*P*	Regression Equation
Peak of acceleration	−0.587	.0001	*Y* = 27.9 − 0.308 *X*
Height of acceleration-1	0.760	<.0001	*Y* = −1.68 + 0.038 *X*
Height of acceleration-2	0.760	<.0001	*Y* = .0.314 + 0.069 *X*
Width of acceleration-1	−0.407	.0124	*Y* = 14.8 − 0.219 *X*
Width of acceleration-2	−0.353	.0320	*Y* = 13.6 − 0.039 *X*
Peak of velocity	−0.508	.013	*Y* = 20.2 − 0.136 *X*
Height of velocity	0.547	.0005	*Y* = −1.19 + 0.058 *X*
Width of velocity	−0.372	.0235	*Y* = 14.0 − 0.048 *X*
Peak of ½ fibrin formation	−0.527	.0008	*Y* = 20.6 − 0.131 *X*
Height of ½ fibrin formation	−0.301	.0699	*Y* = 17.4 − 0.020 *X*
APTT	−0.541	.0006	*Y* = 23.9 − 0.227 *X*
Fibrinogen	0.021	.9027	*Y* = 7.76 + 0.003 *X*

Abbreviation: APTT, activated partial thromboplastin time.

**Table 2.** Parameters of APTT Waveform in Patients With Hemophilia With or Without Inhibitor, Those With VWD, and Healthy Volunteers.

	HV	Hemophilia	Hemophilia With Inhibitor	VWD
Peak of acceleration	35.5 (32.6-37.4)	53.9^a,b^ (44.1-66.5)	112^a,c^ (69.0-120)	46.7^b^ (32.5-59.5)
Height of acceleration-1	457 (411-535)	237^a,d^ (161-354)	25.3^a,e^ (20.9-33.5)	397^b,f^ (360-510)
Height of acceleration-2	224 (170-255)	93.7^a,d^ (57.2-167)	5.01^a,e^ (3.90-5.80)	241^b,c^ (176-257)
Width of acceleration-1	7.45 (6.80-8.00)	20.0^a,g^ (15.7-28.0)	43.2^a,f^ (23.1-88.7)	13.4^a,b^ (10.4-22.1)
Width of acceleration-2	17.7 (16.7-18.7)	49.4^a^ (35.0-195)	210^a^ (40.0-283)	41.8^a^ (35.6-300)
Peak of velocity	38.7 (36.3-40.8)	64.9^a,b^ (52.2-85.0)	154.3^a,c^ (83.8-219)	52.4^b^ (36.8-72.4)
Height of velocity	183 (144-200)	155^d^ (113-217)	27.9^a,e^ (24.7-50.7)	209^b,f,h^ (182-485)
Width of velocity	19.5 (18.0-20.3)	64.0^a^ (50.1-100)	234^a^ (52.9-316)	52.0^a^ (43.4-100)
Peak of ½ fibrin formation	40.9 (37.8-42.7)	66.2^a,b^ (54.4-104)	159^a,c^ (118-223)	53.8^b^ (37.9-73.7)
Height of ½ fibrin formation	242 (207-306)	443^a^ (296-578)	502^i^ (252-870)	302 (182-336)

Abbreviations: APTT, activated partial thromboplastin time; HV, healthy volunteers; VWD, von Willebrand disease.

^a^ *P*< .001 compared with HV.

^b^ *P* < .01 compared with hemophilia with inhibitor.

^c^ *P* < .01 compared with hemophilia.

^d^ *P* < .001 compared with hemophilia with inhibitor.

^e^ *P* < .001 compared with hemophilia.

^f^ *P* < .05 compared with hemophilia.

^g^ *P* < .05 compared with hemophilia with inhibitor.

^h^ *P* < .05 compared with HV.

^i^ *P* < .01 compared with HV.

There were no significant differences in the APTT waveform between patients with hemophilia without inhibitor and patients positive for LA, but the peak of acceleration and ½ fibrin formation were significantly longer in patients with hemophilia with inhibitor than in the patients positive for LA, and the height of acceleration-1 and acceleration-2 and velocity were significant lower in patients with hemophilia with inhibitor than in patients positive for LA (Table 3).

**Table 3.** Parameters of APTT Waveform in Patients With Hemophilia With or Without Inhibitor, Those With VWD, and Patients With LA.

	LA	Hemophilia	Hemophilia With Inhibitor	VWD
Peak of acceleration	64.8 (44.5-79.6)	53.9 (44.1-66.5)	112 (69.0-120)	46.7^a^ (32.5-59.5)
Height of acceleration-1	159 (69.1-411)	237 (161-354)	25.3^b^ (20.9-33.5)	397^a^ (360-510)
Height of acceleration-2	47.6 (34.0-100)	93.7 (57.2-167)	5.01^b^ (3.90-5.80)	241^c^ (176-257)
Width of acceleration-1	26.7 (15.4-38.6)	20.0 (15.7-28.0)	43.2 (23.1-88.7)	13.4 (10.4-22.1)
Width of acceleration-2	47.6 (34.0-100)	49.4 (35.0-195)	210 (40.0-283)	41.8 (35.6-300)
Peak of velocity	85.7 (3.80-165)	64.9 (52.2-85.0))	154.3 (83.8-219)	52.4 (36.8-72.4)
Height of velocity	149 (87.1-215)	155 (113-217)	27.9^c^ (24.7-50.7)	209 (182-485)
Width of velocity	66.3 (41.9-100)	64.0 (50.1-100)	234 (52.9-316)	52.0 (43.4-100)
Peak of ½ fibrin formation	79.0 (49.3-92.0)	66.2 (54.4-104)	159^b^ (118-223)	53.8 (37.9-73.7)
Height of ½ fibrin formation	479 (254-589)	443 (296-578)	502 (252-870)	302 (182-336)

Abbreviations: APTT, activated partial thromboplastin time; LA, lupus anticoagulant; VWD, von Willebrand disease.

^a^ *P* < .05 compared with LA.

^b^ *P* < .01 compared with LA.

^c^ *P* < .001 compared with LA.

The peak times of acceleration and velocity were longer and the width of acceleration-1 of the APTT waveform was greater in patients with hemophilia without an inhibitor than in the patients treated with warfarin (Table 4). The peak times of acceleration and ½ fibrin formation were significantly longer in patients with hemophilia with inhibitor than in patients treated with warfarin. The height of acceleration-1 and acceleration-2 and velocity were significantly lower in patients with hemophilia with an inhibitor than in patients treated with warfarin. Wave changes in the APTT were observed in all 22 patients positive for LA. However, the biphasic wave form was observed in patients with hemophilia with FVIII activity <10.0%. The peak times of acceleration and velocity and the APTT were significantly longer and the height of acceleration-1 and velocity were higher in those with a biphasic wave pattern than in those without (Table 5). There were no significant differences in the parameters of APTT waveform between patients with hemophilia A and B.

**Table 4.** Parameters of APTT Waveform in Patients With Hemophilia With or Without Inhibitor, Those With VWD, and Patients Treated With Warfarin.

	Warfarin	Hemophilia	Hemophilia With Inhibitor	VWD
Peak of acceleration	42.8 (38.0-49.4)	53.9^a^ (44.1-66.5)	112^b^ (69.0-120)	46.7 (32.5-59.5)
Height of acceleration-1	421 (162-499)	237 (161-354)	25.3^b^ (20.9-33.5)	397 (360-510)
Height of acceleration-2	54.0 (46.7-73.2)	93.7 (57.2-167)	5.01^b^ (3.90-5.80)	241^c^ (176-257)
Width of acceleration-1	11.6 (9.10-20.1)	20.0^a^ (15.7-28.0)	43.2^a^ (23.1-88.7)	13.4 (10.4-22.1)
Width of acceleration-2	58.1 (48.2-86.8)	49.4 (35.0-195)	210 (40.0-283)	41.8 (35.6-300)
Peak of velocity	103 (75.4-150)	64.9^a^ (52.2-85.0))	154.3 (83.8-219)	52.4^a^ (36.8-72.4)
Height of velocity	183 (117-216)	155 (113-217)	27.9^c^ (24.7-50.7)	209 (182-485)
Width of velocity	72.2 (61.1-99.6)	64.0 (50.1-100)	234 (52.9-316)	52.0 (43.4-100)
Peak of ½ fibrin formation	51.9 (44.3-88.5)	66.2 (54.4-104)	159^b^ (118-223)	53.8 (37.9-73.7)
Height of ½ fibrin formation	246 (188-317)	443^b^ (296-578)	502^a^ (252-870)	302 (182-336)

Abbreviations: APTT, activated partial thromboplastin time; VWD, von Willebrand disease; Warfarin, patients treated with warfarin.

^a^ *P* < .05 compared with patients treated with warfarin.

^b^ *P* < .01 compared with patients treated with warfarin.

^c^ *P* < .001 compared with patients treated with warfarin.

**Table 5.** Parameters of Aptt Waveform Between Biphasic Pattern Positive and Negative of Patients With Hemophilia, and Those With VWD.

	Biphasic Pattern		*P* Value
	Positive	Negative	
Peak of acceleration (seconds)	65.1 (54.7-86.0)	40.1 (32.5-41.4)	<.001
Height of acceleration-1	161 (38.8-240)	462 (360-838)	<.001
Peak of velocity (seconds)	78.1 (67.3-125)	44.1 (36.8-50.0)	<.001
Height of velocity	123 (53.5-184)	199 (177- 368)	<.05
FVIII (seconds)	2.10 (0.50-2.98)	20.9 (9.20-38.1)	<.001
APTT (seconds)	61.3 (53.8-102)	40.4 (38.8-42.1)	<.001

Abbreviations: APTT, activated partial thromboplastin time; VWD, von Willebrand disease.

## Discussion

Changes in the APTT waveform have been reported to predict coagulation factor deficiency or LA positivity.^6^ In the present study, regarding the APTT waveform, the peak times of acceleration, velocity, and ½ fibrin formation and the width of acceleration and velocity were significantly longer and the height of acceleration was significantly lower in patients with hemophilia than in healthy volunteers. The APTT is now used to evaluate patients with hemophilia A. The APTT value is determined by the peak time of ½ fibrin formation (CS-2500) or acceleration-1 of APTT waveform (ACL-TOP). Our study showed that the peak time of acceleration-1 had slightly better correlation with FVIII activity than that of ½ fibrin formation. Furthermore, the height of acceleration-1 and acceleration-2 was significantly correlated with the FVIII activity, suggesting that the height of acceleration in the APTT waveform is more useful for monitoring hemophilia A than a routine APTT assay. The results of APTT waveform analyses are reported to be well correlated with the FVIII activity and to be useful for the management of hemophilia.^13–15^

The height of the velocity of the APTT waveform in patients with hemophilia was within normal range, suggesting that the start of coagulation was markedly late but the velocity of coagulation was not late in hemophilia. In addition, the height of both acceleration and velocity was significantly lower in patients with hemophilia A with inhibitor than in healthy volunteers, suggesting that the decreased height of acceleration and velocity were caused by reduced FVIII activity; indeed, the FVIII activity was markedly lower in patients with hemophilia A with inhibitor than in those without inhibitor. Although patients with AHA have been reported to be associated with severe bleeding,^16^ the present study was unable to clarify the hemostatic abnormalities in AHA. Although our study was small size, there were no significant differences in the APTT analysis findings.

There were no significant differences in the APTT waveform between patients with hemophilia and those positive for LA. In the APTT assay, phospholipids increased the clotting speed, and LA decreases the height of the acceleration and velocity and prolongs the peak times of the acceleration, velocity, and ½ fibrin formation. Therefore, the differential diagnosis between hemophilia and LA can be difficult based solely on the APTT waveform, but that between hemophilia with inhibitor and LA is relatively easy using the APTT waveform. A clotting analysis without lipids such as assessing the thrombin generation time by low-dose tissue factor may be useful for the differential diagnosis between patients with hemophilia and LA.

The biphasic waveform has recently garnered attention in the analysis of the APTT waveform.^5,17,18^ A biphasic wave change in the APTT was observed in all 22 patients positive for LA as well as in patients with hemophilia with FVIII activity <10.0%, which may equate to an APTT >50 seconds. An analysis of the APTT waveform showed a biphasic waveform in 1% FVIII-deficient plasma but not in 10% FVIII-deficient plasma. This suggests that patients with a biphasic waveform and APTT <50 seconds (FVIII activity >10%) may be considered positive for LA. In addition, a biphasic waveform and prolongation of the peak time were not observed in patients treated with warfarin, but a decreased height and an increased width of the acceleration and velocity in the APTT waveform were observed in those patients, suggesting that multiple coagulation factors reduced in patients treated with warfarin. The APTT waveform may not only help diagnose a bleeding tendency but also monitor the course of anticoagulant therapy.

In conclusion, the APTT waveform analysis may be useful for the diagnosis of patients with hemophilia with or without inhibitor, patients with AHA, and patients positive for LA. The height of the acceleration in the APTT waveform reflects the FVIII activity.
